# Enhanced recovery after surgery compliance and postoperative outcomes after liver resection: retrospective cohort study

**DOI:** 10.1093/bjsopen/zrag066

**Published:** 2026-08-11

**Authors:** Valerie Isabel Nottberg, Eleanor Nottberg, Victoria van Rüth, Freya Brodersen, Alina S Ritter, Jan Bardenhagen, Julia Auinger, Parisa Moll-Khosrawi, Jakob R Izbicki, Maximilian Bockhorn, Thilo Hackert, Tarik Ghadban, Asmus Heumann

**Affiliations:** Department of General, Visceral and Thoracic Surgery, University Medical Center Hamburg-Eppendorf, Hamburg, Germany; Department of Urology, Medical Faculty, Heinrich Heine University Düsseldorf, Düsseldorf, Germany; Department of General, Visceral and Thoracic Surgery, University Medical Center Hamburg-Eppendorf, Hamburg, Germany; Department of General, Visceral and Thoracic Surgery, University Medical Center Hamburg-Eppendorf, Hamburg, Germany; Department of General, Visceral and Thoracic Surgery, University Medical Center Hamburg-Eppendorf, Hamburg, Germany; Department of General, Visceral and Thoracic Surgery, University Medical Center Hamburg-Eppendorf, Hamburg, Germany; Department of Anesthesiology, University Medical Center Hamburg-Eppendorf, Hamburg, Germany; Department of Anesthesiology, University Medical Center Hamburg-Eppendorf, Hamburg, Germany; Department of General, Visceral and Thoracic Surgery, University Medical Center Hamburg-Eppendorf, Hamburg, Germany; Department of General and Visceral Surgery, University Medical Center Oldenburg, Oldenburg, Germany; Department of General, Visceral and Thoracic Surgery, University Medical Center Hamburg-Eppendorf, Hamburg, Germany; Department of General, Visceral and Thoracic Surgery, University Medical Center Hamburg-Eppendorf, Hamburg, Germany; Department of General, Visceral and Thoracic Surgery, University Medical Center Hamburg-Eppendorf, Hamburg, Germany

**Keywords:** ERAS, postoperative complication, length of hospital stay, nasogastric tube, mobilization

## Abstract

**Background:**

Enhanced recovery after surgery (ERAS) pathways improve postoperative outcomes across surgical specialties, but the impact of ERAS compliance on outcomes after liver resection remains unclear. A retrospective cohort study of consecutive patients undergoing liver resection in an ERAS-certified centre was conducted to examine the association between overall and item-specific ERAS compliance and postoperative morbidity and length of hospital stay (LOS).

**Methods:**

This retrospective cohort included elective liver resections performed between 2020 and 2024 at an ERAS-certified centre of excellence. ERAS compliance was prospectively recorded and categorized as high (≥ 70%) *versus* low/moderate (< 70%). Primary outcomes were any postoperative complication, major morbidity (Clavien–Dindo grade ≥ IIIa), and LOS; these were assessed by univariable and multivariable analyses, including a sensitivity analysis using an early compliance score excluding postoperative items.

**Results:**

Data from 637 consecutive liver resections were analysed. Mean (standard deviation) overall ERAS compliance was 64.0 (10.2%) and remained stable over time. High compliance was associated with fewer complications (51% (104) *versus* 69.8% (303); odds ratio (OR) 0.45; 95% confidence interval (c.i.) 0.30 to 0.67; *P* < 0.001), lower major morbidity (7.25% (15) *versus* 12.8% (55); OR 0.25, 95% c.i. 0.10 to 0.64; *P* = 0.001), and shorter LOS (7.42 *versus* 10.62 days; mean difference −3.21 days; *P* < 0.001). In multivariable analyses, high overall compliance was associated with major morbidity (OR 0.16; 95% c.i. 0.04 to 0.56; *P* = 0.005). Minimally invasive surgery independently decreased overall complications (OR 0.41; 95% c.i. 0.21 to 0.75; *P* = 0.005), major morbidity (OR 0.13; 95% c.i. 0.01 to 0.66; *P* = 0.050), and LOS (β = −2.31 days; *P* = 0.020). In sensitivity analyses, high compliance remained associated with fewer complications (OR 0.62; 95% c.i. 0.40 to 0.96; *P* = 0.035). Higher intraoperative intravenous fluid volume independently predicted morbidity and prolonged LOS (*P* < 0.001).

**Conclusion:**

*Higher ERAS compliance after liver resection is associated with lower severe morbidity and shorter LOS.* Prioritizing potentially high-impact items may maximize recovery benefits.

## Teaser

Enhanced recovery after surgery (ERAS) compliance after liver resection was evaluated in a retrospective cohort of 637 elective resections with prospective ERAS Interactive Audit System documentation. High overall compliance (≥ 70%) was linked to fewer complications, lower major morbidity, and a shorter length of hospital stay; sensitivity analyses excluding postoperative items yielded consistent findings. These results support ERAS audit-and-feedback approaches and identify pathway elements that track with improved recovery after liver resection.

## Introduction

Enhanced recovery after surgery (ERAS) pathways have become an integral component of modern perioperative management in visceral surgery. Initially established in colorectal procedures, ERAS protocols have since demonstrated benefits across various surgical domains, including reduced postoperative complications, shorter hospital stays, and improved patient satisfaction^1–3^. In recent years, the application of ERAS principles has extended to hepatopancreatobiliary surgery, yet the evidence in hepatic surgery remains less systematic^4–7^.

Liver surgery presents unique challenges to the implementation of ERAS protocols due to the heterogeneity of procedures, the variable extent of resection, and the potential for significant intraoperative blood loss and fluid shifts. Nevertheless, retrospective studies indicate that ERAS protocols can be safely and effectively applied to hepatic resections^8–10^. Specifically, recent analyses suggest that compliance with ERAS protocols has emerged as an important correlate of postoperative morbidity and length of hospital stay (LOS). Key measures include early mobilization, restrictive fluid management, and avoidance of unnecessary drains or nasogastric tubes^11^.

Although the clinical value of ERAS components is widely accepted, overall protocol compliance has emerged as a critical determinant of patient outcome: a compliance threshold of ≥ 75% was associated with significantly fewer major complications and reduced LOS in liver surgery^8^, and achieving nine or more ERAS components by postoperative day 3 led to improved clinical outcomes^9^. Institutional learning curves and procedure complexity continue to limit high compliance rates, with major hepatic resections being most affected^12^.

The aim of this study was to investigate the outcome relevance of ERAS protocol implementation in patients undergoing liver resections, analysing the correlation between single ERAS items and overall complications with postoperative morbidity.

## Methods

### Study design and setting

This retrospective observational cohort study was conducted at the University Medical Centre Hamburg Eppendorf, a tertiary referral centre for hepatobiliary surgery and ERAS centre of excellence. The ERAS protocol for liver surgery was implemented institution-wide in January 2020, and compliance with individual ERAS measures was documented prospectively in a dedicated quality registry (ERAS Interactive Audit System; www.erassociety.org; ENCARE, Krista, Sweden). Patients were also registered if preoperative counselling was not completed. Thirty-day follow-up was performed by the ERAS nurse using a structured telephone assessment. All demographic, clinical, and perioperative variables, including ERAS compliance, were extracted in December 2024.

Ethics approval for the study was granted by the University Medical Centre Hamburg Eppendorf’s ethics committee (PV2024-101305-PA-ff), and the study was performed in accordance with the Declaration of Helsinki. This study was conducted in compliance with the STROCSS guidelines for reporting cohort studies in surgery^13^.

### Patient selection

This retrospective cohort included all consecutive adult patients undergoing elective liver resection between January 2020 and December 2024 within a standardized ERAS programme. Seven patients (1.1%) were coded as urgent in administrative data despite meeting non-emergency eligibility criteria and were retained in the cohort. At programme enrolment, patients provided written general consent. For temporal comparison and to evaluate protocol implementation over time, patients were stratified into two periods: 2020–2022 and 2023–2024.

For outcome analyses, overall ERAS compliance was dichotomized at 70% to define high *versus* low compliance, in line with established ERAS literature across surgical specialties and recent liver surgery compliance studies^8,11,14^. According to the Brisbane nomenclature^15^, major hepatectomies were defined as the resection of three or more contiguous liver segments.

### ERAS protocol and compliance definition

The ERAS protocol implemented at the study centre was based on international ERAS^®^ Society guidelines^3^ for liver surgery and included preoperative, intraoperative, and postoperative elements (*Tables S1* and *S2*). Compliance was assessed for each patient and calculated as the percentage of fulfilled measures relative to the total number of applicable items. Compliance was analysed both globally and separately for each perioperative phase. LOS was defined as the number of days from the date of admission to the date of discharge. Readmissions were captured throughout the study period. Any readmission within 30 days after the index surgery was incorporated by adding the duration of the readmission to the index LOS. Key elements included patient education, preoperative carbohydrate loading, restrictive fluid therapy, avoidance of routine drains and nasogastric tubes, early mobilization from postoperative day 0, and early initiation of oral nutrition. Primary outcomes were postoperative complications (Clavien–Dindo grade ≥ I), major complications (Clavien–Dindo grade ≥ IIIa), and LOS. Secondary outcomes included organ-specific complications, such as posthepatectomy liver failure, delayed gastric emptying, and bile leak as defined by current consensus classifications^16–18^.

### Statistical analysis

Statistical analyses were performed in R^®^ version 4.3.3 (R Foundation for Statistical Computing, Vienna, Austria). Data are presented as *n*/*N* (%) and as the mean with standard deviation (s.d.) or median with interquartile range (i.q.r.), as appropriate. Group comparisons used *t* tests or the Mann–Whitney *U* test for continuous variables and χ^2^ tests or Fisher’s exact test for categorical variables. Associations between ERAS compliance and outcomes were assessed using logistic regression (binary endpoints) and linear regression for LOS, reporting odds ratios (ORs) or β coefficients with their 95% confidence interval (c.i.). Multivariable models included covariates selected by univariable screening (*P* < 0.1) and *a priori* clinical relevance, with key confounders retained regardless of univariable results. To reduce potential reverse causality, sensitivity analyses used an early compliance score including preoperative and intraoperative components only (excluding nasogastric tube use, mobilization, and oral intake), adjusted for age, American Society of Anesthesiologists grade, surgical approach, and intraoperative intravenous fluid volume. Two-sided *P* < 0.05 was considered statistically significant.

## Results

### Patient characteristics

Between 2020 and 2024, 637 adult patients underwent elective liver resection patients and were included in the study (2020–2022, 417; 2023–2024, 220). Missing covariate data affected 72 of the 637 patients (11.3%); complete case analyses were performed without multiple imputation. The overall median age was 62 (i.q.r. 52–72) years with an older population in 2023–2024 (*P* < 0.05). Malignancy was the indication in 69.2% of patients (441) and was stable over time (*P* = 0.652). Wedge/other minor (36.9%, 235) and segmental (34.5%, 220) resections predominated. Overall, major hepatectomies accounted for 22% (140) of procedures (*Table 1*).

**Table 1:** Baseline characteristics of patients undergoing liver resection in 2020–2022 and 2023–2024

	2020–2022 (*n* = 417)	2023–2024 (*n* = 220)	Total (*n* = 637)	P*
Age (years), median (i.q.r.)	61.0 (51.0–71.0)	64.0 (54.3–73.6)	62.0 (52.0–72.0)	0.045
**Sex**				0.559
Female	192 (46.0%)	107 (48.6%)	299 (46.9%)	
Male	225 (54.0%)	113 (51.4%)	338 (53.1%)	
BMI (kg/m^2^), mean(s.d.)	25.8(5.2)	27.5(5.75)	26.44(5.50)	< 0.001
ASA grade ≥ II	219 (52.5%)	134 (60.9%)	353 (55.4%)	0.074
**Smoking status**				0.823
Yes	83 (19.9%)	44 (20.0%)	127 (19.9%)	
Diabetes	59 (15.0%)	42 (19.1%)	101 (15.9%)	0.258
**Alcohol use**				0.344
Yes	76 (18.2%)	31 (14.1%)	107 (16.8%)	
WHO Class ≥ II	36 (8.6%)	8 (3.6%)	44 (6.9%)	0.020
Immunosuppression	10 (2.4%)	3 (1.4%)	13 (2.0%)	0.558
Neoadjuvant chemotherapy	64 (15.4%)	32 (14.6%)	96 (15.1%)	0.948
Neoadjuvant radiotherapy	9 (2.2%)	5 (2.3%)	14 (2.2%)	0.948
Previous abdominal surgery	229 (54.9%)	91 (41.4%)	320 (50.2%)	0.001
Elective procedure	414 (99.3%)	216 (98.2%)	630 (98.9%)	0.268
Operative time (min), mean(s.d.)	234.6(105.1)	198.5(140.8)	222.1(119.8)	< 0.001
Malignancy	286 (68.6%)	155 (70.5%)	441 (69.2%)	0.652
**Main procedure**				0.240
Minor hepatectomy (total)	328 (78,6%)	167 (75,9%)	495 (77.7%)	
Wedge/other minor resection	151 (36.2%)	84 (38.2%)	235 (36.9%)	
Segmentectomy	144 (34.5%)	76 (34.6%)	220 (34.5%)	
Other	11 (2.6%)	3 (1.4%)	14 (2.2%)	
Bile duct resection	3 (0.7%)	0 (0.0%)	3 (0.5%)	
Bisegmentectomy	19 (4.6%)	4 (1.8%)	23 (3.6%)	
**Major procedure (total)**	87 (20.8%)	53 (24.1%)	140 (22%)	0.248
Major hepatectomy (total)				
Extended right hemihepatectomy	27 (6.5%)	12 (5.5%)	39 (6.1%)	
Left hemihepatectomy	23 (5.5%)	13 (5.9%)	36 (5.7%)	
Right hemihepatectomy	26 (6.2%)	24 (10.9%)	50 (7.9%)	
Extended left hemihepatectomy	11 (2.6%)	4 (1.8%)	15 (2.4%)	
Explorative laparotomy without liver resection	2 (0.5%)	0 (0.0%)	2 (0.3%)	
**Additional procedures**				0.144
None	356 (85.4%)	170 (77.3%)	526 (82.6%)	
Colorectal resection	7 (1.7%)	4 (1.8%)	11 (1.7%)	
Major debulking	2 (0.5%)	1 (0.5%)	3 (0.5%)	
Other†	31 (7.4%)	22 (10.0%)	53 (8.3%)	
Other organ resection‡	5 (1.2%)	2 (0.9%)	8 (1.3%)	
**Surgical approach**				< 0.001
Open	260 (62.3%)	95 (43.2%)	355 (55.7%)	
Robotic	77 (18.5%)	31 (14.1%)	108 (17.0%)	
Laparoscopic	64 (15.4%)	78 (35.5%)	142 (22.3%)	
Conversion to open	16 (3.8%)	16 (7.3%)	32 (5.0%)	

Values are *n* (%) unless otherwise stated. †For example cholecystectomy, lymphadenectomy. ‡Splenectomy, hysterectomy. i.q.r., interquartile range; BMI, body mass index; s.d., standard deviation; ASA, American Society of Anesthesiologists; WHO, World Health Organization;, min, minutes. *Welch two sample *t*-test.

Operative approach shifted over time (*P* < 0.001), with open surgery decreasing and laparoscopy increasing (*Table 1*). Postoperative complications were common, with infectious, respiratory, and biliary events predominating (*Table S3*). Of the 107 patients undergoing major hepatectomy, 71% (76) experienced minor or major complications (Clavien–Dindo grade ≥ I), whereas the complication rate after minor hepatectomies (417) was 35.5% (148; OR 0.225, 95% c.i. 0.136 to 0.365; *P* < 0.001).

### Overall and phase-specific ERAS compliance

The mean(s.d.) overall ERAS compliance for the entire cohort was 64.02(10.19)%. Overall compliance did not differ significantly between the two study periods (*P* = 0.818; *Table 2*). The highest individual compliance observed was 89.47%. The proportion of patients with high compliance (≥ 70%) over the study period increased from 30.3% to 35.6% (*P* = 0.178). Postoperative compliance showed greater variability, with failure to meet early mobilization targets (71%, 307) and delayed diet initiation (21%, 91) as the most frequent reasons (*Table 2*). Although overall adherence remained stable, several individual ERAS elements showed significant changes over the study period: preoperative biliary drainage was performed less frequently in the later cohort (4.9% (8) *versus* 8.4% (34); *P* = 0.044); and early mobilization improved, with documentation of mobilization on the day of surgery increasing from 25.2% (105) to 35.9% (79; *P* = 0.013). As indicated in *Table 2*, the overall rate of major morbidity (Clavien–Dindo grade ≥ IIIa) was 10.8% (69). Patients with high preoperative ERAS compliance (≥ 70%) had a higher baseline risk profile (*Table S4*).

**Table 2:** Perioperative enhanced recovery after surgery compliance and individual pathway items

	2020–2022 (*n* = 417)	2023–2024 (*n* = 220)	Total (*n* = 637)	P*
Preadmission education	319 (76.5%)	159 (72.3%)	478 (75.0%)	0.245
Preoperative biliary drainage	34 (8.4%)	8 (4.9%)	42 (6.6%)	0.044
Preoperative oral carbohydrate loading	162 (38.9%)	104 (47.3%)	266 (41.8%)	0.060
Antibiotic prophylaxis	415 (99.5%)	217 (98.6%)	632 (99.2%)	0.348
PONV prophylaxis	388 (93.1%)	191 (86.8%)	579 (90.9%)	0.231
Normothermia	305 (73.1%)	176 (80.0%)	484 (76.0%)	0.069
Intraoperative IV volume (ml), mean(s.d.)	4671.0(3761.1)	4256.4(7078.6)	4529.1(3545.3)	0.150
Avoidance of abdominal drain	55 (13.2%)	14 (6.4%)	69 (10.8%)	0.130
Epidural anaesthesia	175 (42.0%)	101 (45.9%)	276 (43.3%)	0.436
Thromboprophylaxis	413 (99.0%)	168 (76.4%)	581 (91.2%)	0.410
Postoperative NG tube removal (while extubated)	410 (98.3%)	211 (96.4%)	621 (97.5%)	0.112
Gastric stimulation	405 (97.4%)	211 (96.4%)	616 (96.7%)	0.560
Regular diet after surgery	379 (91.1%)	154 (70.3%)	533 (83.7%)	< 0.001
**Mobilization**				
Day of surgery	105 (25.2%)	79 (35.9%)	184 (28.9%)	0.013
Postoperative day 1	361 (86.6%)	197 (89.6%)	558 (87.7%)	0.221
Postoperative day 2	382 (91.6%)	199 (90.5%)	581 (92.3%)	0.029
Postoperative day 3	390 (93.5%)	198 (90.0%)	588 (92.3%)	0.014
Overall perioperative compliance (%), mean(s.d.)	64.11(9.39)	63.86(11.56)	64.02(10.19)	0.818
High compliance > 70 (%), mean(s.d.)	30.3(2.3)	35.6(3.2)	32.9(2.1)	0.178
Preadmission compliance (%), mean(s.d.)	69.38(31.88)	66.16(32.02)	68.27(31.94)	0.154
Preoperative compliance (%), mean(s.d.)	65.63(12.63)	64.50(17.54)	65.24(14.51)	0.363
Intraoperative compliance (%), mean(s.d.)	64.99(13.28)	64.10(11.53)	64.68(12.70)	0.790
Postoperative compliance (%), mean(s.d.)	62.91(15.42)	63.59(19.55)	63.14(16.95)	0.160
Major morbidity (CD ≥ IIIa)	40 (9.6%)	29 (13.2%)	69 (10.8%)	0.072

Values are *n* (%) unless otherwise stated. PONV, postoperative nausea and vomiting; s.d., standard deviation; IV, intravenous; NG, nasogastric; CD, Clavien–Dindo. *Welch two sample *t*-test.

### ERAS compliance and postoperative outcomes

The incidence of postoperative complications during the index hospital stay was 51% (95% c.i. 46.5% to 55.5%; 104) in the high-compliance group, compared with a significantly higher rate of 69.78% (95% c.i. 61.4% to 77.3%; 303) in the low/moderate compliance group (OR 0.45; 95% c.i. 0.30 to 0.67; *P* < 0.001), with statistical significance reached only in minor hepatectomy (OR 0.54; 95% c.i. 0.33 to 0.86; *P* = 0.008; *Table 3*).

**Table 3:** Enhanced recovery after surgery compliance, complication rates, and postoperative outcomes

Outcome	High compliance	Low/moderate compliance	Odds ratio (95% c.i.)	P*
**Postoperative complications, *n* (%), (95% c.i.)**				
Overall	51.0% (46.5 to 55.5)	69.8% (61.4 to 77.3)	0.45 (0.30 to 0.67)	**<0.001**
Major hepatectomy	13 of 21 (61.9%)	63 of 86 (73.3%)	0.60 (0.20 to 1.89)	0.42
Minor hepatectomy	38 of 142 (26.8%)	100 of 247 (40.5%)	0.54 (0.33 to 0.86)	**0.008**
**Major morbidity (Clavien–Dindo ≥IIIa), *n* (%), (95% c.i.)**				
Overall	7.3% (2.4 to 16.1)	12.8% (10.2 to 27.3)	0.25 (0.10 to 0.64)	**0.001**
Major hepatectomy	2 of 21 (9.5%) (1.2 to 30.4)	23 of 86 (26.7%) (17.9 to 37.9)	0.29 (0.03 to 1.37)	0.15
Minor hepatectomy	6 of 142 (4.2%) (1.6 to 9.0)	18 of 247 (7.3%) (4.3 to 11.3)	0.56 (0.18 to 1.52)	0.28

Length of hospital stay, mean days			Mean difference (95% c.i.)	
Overall	7.42	10.6	−3.21 (−4.68 to −1.73)	**<0.001**
Major hepatectomy	12.4	14.0	—	0.40
Minor hepatectomy	6.2	8.0	—	**<0.001**

Reference category = low/moderate compliance. Bold values indicate *P* ≤ 0.05. *Complication and morbidity comparisons: χ^2^ test; length of stay: Welch two-sample *t* test (overall *t* = 4.28).

Major complications (Clavien to Dindo grade ≥ IIIa) were less frequent in the high-compliance group (*P* = 0.001; *Table 3*). In sensitivity analyses, high early ERAS compliance (≥ 70%) remained associated with fewer complications (OR 0.62; 95% c.i. 0.40 to 0.96; *P* = 0.035), whereas overall compliance including postoperative items showed a similar direction but did not reach statistical significance (OR 0.70; 95% c.i. 0.46 to 1.06; *Table S5*).

### Impact of ERAS compliance and individual elements on postoperative outcomes

In univariable analysis, both minimally invasive surgery (*P* < 0.001) and same-day mobilization (*P* < 0.001) were associated with significantly lower overall (*P* = 0.014) and major complication rates (*P* = 0.008). Intraoperative drain placement (*P* < 0.001), postoperative nasogastric tube maintenance (*P* = 0.001), and greater intraoperative intravenous fluid volumes (*P* < 0.001) were linked to increased overall and severe morbidity, with each additional 1 l of fluid corresponding to approximately 58% higher odds of complications. High overall compliance (*P* = 0.003), intraoperative compliance (*P* = 0.008), and postoperative compliance (*P* = 0.035) were associated with reduced major morbidity (Clavien–Dindo grader ≥ IIIa; *Table S6*). Univariable associations between individual ERAS elements and LOS are presented in *Table S6*.

In the multivariable logistic regression model (565 patients), overall perioperative ERAS compliance ≥ 70% was not independently associated with a reduction in overall postoperative complications (*P* = 0.920). Among individual ERAS elements, minimally invasive surgery was a strong independent protective factor (*P* = 0.005), whereas postoperative nasogastric tube placement was associated with complications (*P* = 0.021). Higher intraoperative intravenous fluid volumes were also independently associated with increased morbidity (*P* < 0.001). For major morbidity (Clavien–Dindo grade ≥IIIa), high perioperative compliance ≥ 70% was significantly protective (*P* = 0.005), as was preadmission compliance ≥ 70% (*P* = 0.040). In contrast, preoperative compliance ≥ 70% was associated with an increased risk of severe morbidity (OR 2.55; 95% c.i. 1.08 to 5.87; *P* = 0.020). Minimally invasive surgery again showed a protective effect (*P* = 0.05), and intraoperative fluid overload remained a strong risk factor (*P* < 0.001). Regarding LOS, preadmission compliance ≥ 70% (*P* = 0.018), postoperative nausea and vomiting prophylaxis (*P* < 0.001), minimally invasive surgery (*P* = 0.020), and early mobilization on postoperative day 0 (*P* = 0.005) were independently associated with a shorter LOS. Higher intraoperative intravenous fluid volumes significantly prolonged hospitalization (*P* < 0.001; *Table 4*).

**Table 4:** Multivariable analyses of predictors for overall complications, major morbidity (Clavien–Dindo grade ≥IIIa), and postoperative LOS

	Overall complications		Major morbidity		LOS	
	Odds ratio	*P*	Odds ratio	*P*	β	*P*
Perioperative compliance ≥ 70%	1.04 (0.51, 2.11)	0.920	0.16 (0.04, 0.56)	0.005	−1.45,	0.232
Preadmission compliance ≥ 70%	1.60 (0.86, 2.98)	0.139	0.47 (0.23, 1.01)	0.040	−2.47	0.018
Preoperative compliance ≥ 70%	0.62 (0.33, 1.14)	0.126	2.55 (1.08, 5.87)	0.020	+0.71	0.193
Intraoperative compliance ≥ 70%	0.64 (0.37, 1.09)	0.101	1.17 (0.47, 2.72)	0.719	+0.21	0.826
Postoperative compliance ≥ 70%	0.92 (0.50, 1.71)	0.801	1.45 (0.58, 3.47)	0.400	+1.27	0.220
Preadmission education	0.88 (0.44, 1.76)	0.726	1.94 (0.78, 4.87)	0.150	+1.46	0.217
Preoperative carbohydrate load			1.81 (0.93, 3.54)	0.070	+0.53	0.503
PONV prophylaxis	0.75 (0.37, 1.53)	0.428	0.45 (0.19, 1.16)	0.080	−4.82	< 0.001
Minimally invasive surgery	0.41 (0.21, 0.75)	0.005	0.13 (0.01, 0.66)	0.050	−2.31	0.020
Normothermia			0.96 (0.52, 1.81)	0.886	−0.07	0.927
Early mobilization POD0	0.59 (0.34, 1.01)	0.060	0.78 (0.32, 1.80)	0.573	−2.63	0.005
Postoperative NG tube	18.40 (2.02, 422.2)	0.021	0.77 (0.15, 3.19)	0.736	+1.60	0.206
Drain placement	1.16 (0.52, 2.66)	0.726	1.82 (0.43, 12.78)	0.471	+3.59	0.126
Intraoperative IV volume*	1.05 (1.04, 1.06)	< 0.001	1.02 (1.01, 1.03)	< 0.001	+0.11	< 0.001

Values in parentheses are 95% confidence intervals. *Effect per millilitre of intraoperative IV volume administered; larger volumes are associated with increased risk and prolonged LOS. β, regression coefficient (linear model); LOS, length of hospital stay; PONV, postoperative nausea and vomiting; POD, postoperative day; NG, nasogastric; IV, intravenous.

## Discussion

In this large single-centre cohort of 637 patients, high adherence to the ERAS pathway was associated with reduced major morbidity and a shorter LOS. Achieving ≥ 70% overall compliance was linked to fewer major complications and shorter hospitalization. Although point estimates suggested lower complication rates with high ERAS compliance in both strata, significance was reached only in minor resections, implying that effects may be less pronounced in major hepatectomy, where procedural complexity and baseline risk dominate outcomes; further multicentre studies are warranted to clarify the effects of the ERAS protocol in this subgroup. Consistent with published ERAS studies in liver surgery^5,8–12,19–22^, higher protocol compliance was generally associated with fewer complications and shorter LOS. This study adds to the evidence base by combining prospective ERAS Interactive Audit System-based compliance auditing with phase-specific and item-level analyses in a large liver resection cohort, including procedure complexity subgroup analyses. These findings suggest that both overall ERAS compliance and specific high-impact elements are potential key determinants of recovery after liver surgery. Minimally invasive surgery, early mobilization, avoidance of postoperative nasogastric tubes, avoidance of drains, and optimized fluid management were most associated with improved outcomes. An independent benefit of minimally invasive surgery was confirmed, which was associated with a 59% reduction in overall morbidity and a mean reduction in LOS of > 2 days^23^. The rise in minimally invasive approaches over the study period suggests a synergistic relationship between ERAS principles and evolving surgical techniques. High intraoperative intravenous fluid volumes were independently associated with higher complication rates and prolonged hospitalization, supporting recent meta-analyses^24^ advocating for goal-directed fluid therapy in hepatobiliary procedures. Although the role of same-day mobilization remains debated^25^, the present data demonstrate an independent benefit. Using an early compliance score restricted to preoperative and intraoperative elements reduced the risk of reverse causality from postoperative items. Preoperative compliance ≥ 70% was associated with increased major morbidity (*P* = 0.020), best interpreted as a marker of case complexity and selective intensification of preoperative optimization rather than a causal effect. Apparent non-compliance with elements such as omission of prophylactic abdominal drains may reflect patient selection and operative strategy (for example, cholestasis and biliary drainage) rather than true deviation from the ERAS pathway. Preoperative oral carbohydrate loading and normothermia maintenance did not show a measurable association with major morbidity, possibly reflecting high baseline adherence and limited incremental contrast, or a less pronounced impact on hepatobiliary outcomes compared with other surgical contexts^26,27^. The proportion of patients with high compliance (≥ 70%) increased from 30.3% (126) to 35.6% (78) over the study period (*P* = 0.178), reflecting an institutional learning curve; the highest individual compliance observed was 89.47%, demonstrating that sustained high adherence over a long period is achievable^4,11^. In line with recent hepatopancreatobiliary reports^25^, failure to meet early mobilization targets was the most frequent reason for reduced postoperative compliance (71%, 307). Digital health tools, including mobile applications and wearable activity trackers, may further support ERAS adherence and warrant prospective evaluation^28,29^.

This retrospective observational study does not permit causal inference, and residual confounding and reverse causality cannot be excluded. Item-level associations are prone to confounding by indication and reverse causality (for example, drains, nasogastric tubes, and mobilization reflecting case complexity or early complications) and should be interpreted as markers of clinical course; some estimates were imprecise owing to sparse events. A complete case approach was used and may introduce bias if data were not missing at random. Weight change on postoperative day 1 was not recorded in 60.2% (383) of patients, reducing measurable overall compliance. Major hepatectomy comprised 22% of procedures, limiting power for subgroup analyses and generalizability to major resections. Outcomes were derived from routine documentation, and the single-centre setting in an ERAS-certified high-volume tertiary centre may limit external generalizability.

High compliance (≥ 70%) was associated with fewer postoperative complications and a reduction in LOS of > 2 days. A minimally invasive approach, mobilization on the day of surgery, avoidance of prophylactic abdominal drains, and limited nasogastric tube use showed the strongest associations with improved outcomes, with the most consistent effects observed in minor liver resections.

## Supplementary Material

zrag066_Supplementary_Data

## Data Availability

Deidentified data are available upon reasonable request.
