# Supplementary material for: Enhanced recovery after surgery compliance and postoperative outcomes after liver resection: retrospective cohort study
Source: BJS Open. 2026 Aug 11;10(4):zrag066. doi: 10.1093/bjsopen/zrag066 (PMC13459744; doi:10.1093/bjsopen/zrag066)
Supplement: Supplementary file 1 [file zrag066_supplementary_data.docx]

### **Supplemental Digital Content**

[**Supplemental Table 1**: Local implementation of ERAS items for liver surgery.	2](#_Toc223854916)

[**Supplemental Table 2:** Definition of ERAS items used for compliance calculations.	3](#_Toc223854917)

[**Supplemental Table 3:** Postoperative Course and Complications (n = 637). Bold values indicate p≤0.05.	4](#_Toc223854918)

[**Supplemental Table 4:** Baseline characteristics by preoperative ERAS compliance (<70% vs ≥70%). Values are median (i.q.r.) or n (%). P values from Wilcoxon rank-sum test or χ²/Fisher’s exact test, as appropriate.	5](#_Toc223854919)

[**Supplemental Table 5:** Sensitivity analyses for postoperative complications using overall ERAS compliance versus early ERAS compliance (pre- and intraoperative items only). Values are odds ratios (OR) with 95% confidence intervals from multivariable logistic regression.	6](#_Toc223854920)

[**Supplemental Table 6:** Univariate Analysis: Impact of ERAS Compliance and Individual Components on Postoperative Outcomes. Bold values indicate p≤0.05.	7](#_Toc223854921)

| **ERAS item** | **Local implementation** |
| --- | --- |
| **Preadmission patient education** | Structured counseling by ERAS-trained nurse; patient diary issued; optimization of risk factors (e.g., alcohol, smoking, nutrition) |
| **Preadmission screening and optimization** | PBM protocol: Hb <13 g/dl, Ferritin <100 µg/l, transferrin saturation <20%; nutritional assessment; diabetes control (HbA1c) |
| **Fasting and carbohydrate loading** | 4 × 200 ml carbohydrate drinks until 2h before surgery (except uncontrolled diabetes, gastric outlet obstruction); clear fluids allowed until 2h pre-op |
| **Preemptive analgesia** | Metamizole 1g IV q6h; Paracetamol 1g IV q6h if allergy; epidural catheter only for open resections |
| **Anti-emetic prophylaxis** | Ondansetron 4 mg IV + dexamethasone 4 mg IV at induction |
| **Intraoperative fluid strategy** | Restrictive, near-zero balance; goal-directed if high-risk cases |
| **Anesthesia protocol** | Balanced anesthesia; Sevoflurane ± TIVA; Sufentanil or remifentanil; Rocuronium guided by neuromuscular monitoring |
| **Patient warming** | Forced-air heating and warmed IV fluids |
| **Pneumatic calf compression** | Intermittent pneumatic compression started intraoperatively and continued until 24h postoperatively |
| **Postoperative fluid management** | Restrictive IV strategy; early switch to oral intake POD0–1 whenever tolerated |
| **Postoperative analgesia** | Multimodal: metamizole ± paracetamol ± ibuprofen; epidural for open resections |
| **Drain and line management** | No prophylactic drains in minor resections; selective use for major resections with biliary reconstruction; removal POD3 if output <300 ml. Determination of Bilirubin in drain POD1. |
| **Nasogastric tube** | Not routinely placed; remove directly post-op if inserted |
| **Early mobilization** | POD0: out of bed; POD1: ≥500 steps; POD2: ≥2000 steps; POD3: ≥3000 steps |
| **Diet advancement** | POD0: sips of water; POD1: tea, yoghurt, soup; POD2: soft diet; POD3+: regular diet if tolerated |
| **Discharge criteria** | CRP <80, afebrile, adequate oral intake, mobilization independent, no IV medications |

**Supplemental Table 1: Local implementation of ERAS items for liver surgery. Abbreviations:**ERAS, Enhanced Recovery After Surgery; PBM, patient blood management; Hb, haemoglobin; g/dl, grams per decilitre; µg/l, micrograms per litre; HbA1c, glycated haemoglobin; h, hour(s); pre-op, preoperative; IV, intravenous; q6h, every 6 hours; TIVA, total intravenous anaesthesia; POD, postoperative day; CRP, C-reactive protein.

| **ERAS item** | **Compliance definition** |
| --- | --- |
| **Preadmission counseling** | Completed structured ERAS education |
| **Preoperative carbohydrate loading** | Consumption of ≥400 ml carbohydrate drinks up to 2h pre-op |
| **Smoking cessation** | Abstinence or reduction ≥4 weeks pre-op |
| **Antibiotic prophylaxis** | Administered ≤60 min before incision |
| **PONV prophylaxis** | Ondansetron ± dexamethasone administered at induction |
| **Intraoperative drain avoidance** | No prophylactic drain if no biliary reconstruction |
| **Nasogastric tube avoidance** | No NG tube in place beyond PACU |
| **Normothermia** | Intraop temperature ≥36.0°C |
| **Early mobilization POD0** | Standing or walking on day of surgery |
| **Mobilization POD1–3** | POD1: ≥500 steps; POD2: ≥2000 steps; POD3: ≥3000 steps |
| **Oral intake POD1** | Tea/yoghurt/soup tolerated |
| **Diet advancement POD2+** | Soft or regular diet tolerated without GI intolerance |
| **IV fluid discontinuation** | Switch to oral fluids ≤POD1 if tolerated |
| **Urinary catheter removal** | Removal on POD1 unless contraindicated |
| **Follow-up** | 30-day follow-up completed |

**Supplemental Table 2:** Definition of ERAS items used for compliance calculations.

| **Complication** | **n (%)** | **Subclassification** | **n (%)** |
| --- | --- | --- | --- |
| **Bile leak** | 70 (11.0%) | Grade A | 27 (38.57%) |
|  |  | Grade B | 31 (44.29%) |
|  |  | Grade C | 12 (17.14%) |
| **Post-hepatectomy liver failure (PHLF)** | 14 (2.20%) | Grade A | 8 (57.14%) |
|  |  | Grade B | 6 (42.86%) |
| **Ascites requiring intervention** | 54 (8.48%) | — | — |
| **Biloma** | 14 (2.20%) | — | — |
| **Delayed gastric emptying** | 17 (2.67%) | Grade A | 8 (47.06%) |
|  |  | Grade B | 8 (47.06%) |
|  |  | Grade C | 1 (5.88%) |
| **Respiratory complications** | 63 (9.89%) | — | — |
| **Cardiovascular complications** | 35 (5.49%) | — | — |
| **Infectious complications** | 126 (19.78%) | Wound infection | 46 (7.21%) |
|  |  | Intra-abdominal abscess | 37 (5.81%) |
|  |  | Urinary tract infection | 20 (3.14%) |
|  |  | Sepsis | 12 (1.88%) |
|  |  | Septic shock | 5 (0.78%) |
| **Renal, hepatic, pancreatic, and gastrointestinal complications** | 90 (14.13%) | Renal dysfunction | 48 (7.53%) |
|  |  | Postoperative paralytic ileus | 17 (2.67%) |
| **Psychiatric complications** | 23 (3.61%) | — | — |
| **Major Morbidity (CD** ≥ **IIIa)** | 69 (10.8) |  |  |

**Supplemental Table 3:** Postoperative Course and Complications (n = 637). Bold values indicate p≤0.05. **Abbreviations:** ERAS, Enhanced Recovery After Surgery; h, hour(s); pre-op, preoperative; min, minutes; PONV, postoperative nausea and vomiting; NG, nasogastric; PACU, post-anaesthesia care unit; intraop, intraoperative; POD, postoperative day; GI, gastrointestinal; IV, intravenous.

| **Variable** | **<70% compliance (n=503)** | **≥70% compliance (n=134)** | **p-value** |
| --- | --- | --- | --- |
| **Age, years** | 60 (48–70) | 67 (59–74) | <0.001 |
| **BMI, kg/m²** | 25.45 (22.41–28.72) | 27.17 (24.10–31.24) | <0.001 |
| **ASA physical status** |  |  | 0.002 |
| ASA I | 21 (4.2%) | 0 (0%) |  |
| ASA II | 217 (43.1%) | 41 (30.6%) |  |
| ASA III | 258 (51.3%) | 88 (65.7%) |  |
| ASA IV | 4 (0.8%) | 3 (2.2%) |  |
| Unknown | 3 (0.6%) | 2 (1.5%) |  |
| **Preoperative biliary drainage** |  |  | 0.31 |
| None | 407 (80.9%) | 116 (86.6%) |  |
| Endoscopic drainage | 27 (5.4%) | 6 (4.5%) |  |
| Percutaneous drainage | 9 (1.8%) | 0 (0%) |  |
| **Preoperative nutritional treatment** |  |  | 0.087 |
| Normal diet | 205 (40.8%) | 52 (38.8%) |  |
| Oral immunonutrition | 120 (23.9%) | 22 (16.4%) |  |
| Oral supplements | 161 (32.0%) | 58 (43.3%) |  |
| Parenteral nutrition | 1 (0.2%) | 0 (0%) |  |
| Unknown | 16 (3.2%) | 2 (1.5%) |  |

**Supplemental Table 4:** Baseline characteristics by preoperative ERAS compliance (<70% vs ≥70%). Values are median (i.q.r.) or n (%). P values from Wilcoxon rank-sum test or χ²/Fisher’s exact test, as appropriate. **Abbreviations:** BMI, body mass index; ASA, American Society of Anesthesiologists.

| **Variable** | **Model A Overall ERAS compliance** |  | **Model B Early ERAS compliance** |  |
| --- | --- | --- | --- | --- |
|  | OR | 95% CI | OR | 95% CI |
| **ERAS compliance ≥ 70%** |  |  |  |  |
| Overall compliance | 0.70 | 0.46 – 1.06 | — | — |
| Early compliance (pre- & intraoperative) | — | — | **0.62** | **0.40 – 0.96** |
| Age (per year) | 1.01 | 1.00 – 1.03 | 1.02 | 1.00 – 1.03 |
| **ASA physical status** |  |  |  |  |
| ASA II vs I | 0.80 | 0.27 – 2.77 | 0.85 | 0.28 – 2.95 |
| ASA III vs I | 0.91 | 0.30 – 3.18 | 0.99 | 0.32 – 3.49 |
| ASA IV vs I | 1.14 | 0.13 – 8.84 | 1.22 | 0.14 – 9.45 |
| Unknown vs I | 1.65 | 0.15 – 20.90 | 1.98 | 0.18 – 24.66 |
| **Open vs minimally invasive approach** | **2.42** | **1.60 – 3.70** | **2.25** | **1.46 – 3.48** |
| **Intraoperative IV fluid volume (per 1 L)** | **1.52** | **1.39 – 1.67** | **1.53** | **1.39 – 1.68** |

**Supplemental Table 5:** Sensitivity analyses for postoperative complications using overall ERAS compliance versus early ERAS compliance (pre- and intraoperative items only). Values are odds ratios (OR) with 95% confidence intervals from multivariable logistic regression.

Model A included overall ERAS compliance (≥70%) and was adjusted for age, ASA physical status, surgical approach (open vs minimally invasive) and intraoperative intravenous (IV) fluid volume (per 1 L). Model B used an early ERAS compliance score (≥70%) excluding postoperative items (nasogastric tube use, mobilisation and oral intake) and was adjusted for the same covariates. Patients with missing covariate data were excluded (complete-case analysis); Model A n=626, Model B n=626.

**Abbreviations:** ERAS = Enhanced Recovery After Surgery; ASA = American Society of Anesthesiologists; OR = odds ratio; CI = confidence interval; IV = intravenous.

|  | **Overall Complications (*p*)** | **Major Morbidity CD** ≥ **IIIa (*p*)** | **Length of Stay (days) -Low/High Compliance** | **LOS *p*** |
| --- | --- | --- | --- | --- |
| **Overall ERAS compliance ≥70%** | **0.0001** | **0.0032** | 10.62 / 7.42 | **<0.0001** |
| **Pre-admission compliance** | 0.768 | 0.268 | 10.81 / 9.15 | **0.02** |
| **Preoperative compliance** | 0.134 | 0.758 | 9.96 / 9.77 | 0.83 |
| **Intraoperative compliance** | **<0.0001** | **0.008** | 10.80 / 7.57 | **<0.0001** |
| **Postoperative compliance** | **0.003** | **0.035** | 10.61 / 8.37 | **0.004** |
| **Minimally invasive surgery** | **<0.001** | 0.014 | 10.69 / 6.18 | **<0.0001** |
| **Normothermia** | **0.0364** | 0.728 | 9.32 / 10.33 | 0.170 |
| **Early mobilization POD 0** | **<0.001** | **0.008** | 11.23 / 6.72 | **<0.0001** |
| **Abdominal drain placement** | **<0.001** | **0.048** | 5.54 / 10.49 | **<0.0001** |
| **Postoperative nasogastric tube** | **0.0013** | 0.024 | 9.68 / 19.13 | 0.010 |
| **Intraoperative IV volume (high)** | **<0.001** | 0.167 | — | **<0.001** |
| **Pre-admission education** | 0.728 | 0.611 | 10.05 / 9.88 | 0.858 |
| **Oral carbohydrate loading** | 0.108 | 0.141 | 9.91 / 9.94 | 0.966 |
| **PONV prophylaxis** | 0.686 | 0.325 | 13.93 / 9.52 | **0.022** |

**Supplemental Table 6:** Univariate Analysis: Impact of ERAS Compliance and Individual Components on Postoperative Outcomes. Bold values indicate p≤0.05. **Abbreviations:** ERAS = Enhanced Recovery After Surgery; CD = Clavien–Dindo classification; NS = not significant; POD = postoperative day.
